# Differentiating between Hemorrhagic Infarct and Parenchymal Intracerebral Hemorrhage

**DOI:** 10.1155/2012/475497

**Published:** 2012-04-02

**Authors:** P. M. C. Choi, J. V. Ly, V. Srikanth, H. Ma, W. Chong, M. Holt, T. G. Phan

**Affiliations:** ^1^Stroke and Ageing Research Group, Neurosciences Program, Southern Clinical School, Monash Medical Centre, Monash University, Level 5, Block E, 246 Clayton Road, Clayton, Melbourne, VIC 3168, Australia; ^2^Department of Diagnostic Radiology, Monash Medical Centre, Southern Health, 246 Clayton Road, Clayton, Melbourne, VIC 3168, Australia

## Abstract

Differentiating hemorrhagic infarct from parenchymal intracerebral hemorrhage can be difficult. The immediate and long-term management of the two conditions are different and hence the importance of accurate diagnosis. Using a series of intracerebral hemorrhage cases presented to our stroke unit, we aim to highlight the clues that may be helpful in distinguishing the two entities. The main clue to the presence of hemorrhagic infarct on computed tomography scan is the topographic distribution of the stroke. Additional imaging modalities such as computed tomography angiogram, perfusion, and magnetic resonance imaging may provide additional information in differentiating hemorrhagic infarct from primary hemorrhages.

## 1. Introduction

In acute stroke, the differential diagnosis of hemorrhage detected on computed tomography (CT) scan ranges from hemorrhagic infarct (HI), primary intracerebral hemorrhage (ICH) to hemorrhage from venous infarction. The differentiation between the first two conditions can be difficult, and there are currently no radiological criteria to assist in this regard. It is, therefore, not surprising that previous investigators have found poor agreement in making a diagnosis of HI or ICH [2].

HI, or hemorrhagic transformation of an infarct, occurs in approximately one-third of cases of ischaemic stroke [3]. When an infarct is immediately followed by the occurrence of petechial hemorrhage in the same arterial territory, the diagnosis of HI is easily made. However, when brain imaging is delayed after the onset of the patient's stroke symptoms, an erroneous diagnosis of ICH may be made if the hemorrhage appears confluent on CT. This issue of misdiagnosing HI has been recently raised by other investigators and may also be partly responsible for the overestimation of the prevalence of ICH [4]. Correct assignment of diagnosis is critical in guiding both acute and long-term management and also estimating prognosis. Patients with ischaemic stroke are more likely to develop recurrent ischaemic stroke than ICH. Antiplatelet is the mainstay therapy for this group of patients. Likewise, the finding of HI and atrial fibrillation suggests that the stroke mechanism is cardioembolism and anticoagulation needs to be considered. Furthermore, it has also been suggested that some cases of “ICH” in patients on anticoagulants may in fact be HI and thus represent “failure of anticoagulation” rather than anticoagulant-induced ICH [5].

## 2. Clinical Factors and Mechanism of HI

HI occurs more commonly in elderly patients and those with larger infarcts [6]. Among patients receiving thrombolytic therapy, it occurs more commonly in patients with diabetes and hypertension [7, 8]. It has also been associated with carotid endarterectomy [9] and carotid artery stenting.

HI typically happens within 1-2 weeks after stroke onset, less commonly (~9%) in the first 24 hours [6]. The occurrence of dense hematoma complicating HI may be even lower at approximately 3% [6]. The mechanism of HI has been postulated to be due to breakdown of the basal lamina of microvessels related to activity of matrix metalloproteinase [10]. This may be a consequence of prolonged ischaemia and exacerbated by recanalisation of the occluded artery. It has been suggested that tissue plasminogen activator (tPA) may exacerbate this process, but spontaneous intrainfarct hematoma can also occur in the absence of thrombolysis [11].

## 3. Recognition of HI on CT Scans

A classification of HI based on the topography and intensity of hemorrhage on CT has previously been proposed by Moulin et al. in 1993 [1]: type 1, a multifocal or pethechial hemorrhagic infarction and type 2, an intra-infarct hematoma. The appearance of the latter can mimic ICH on CT scans. Careful observation of the deep structures involved by the stroke lesion and the topography of the surrounding hypodensity may help in reaching the correct diagnosis.

**Figure 1:**
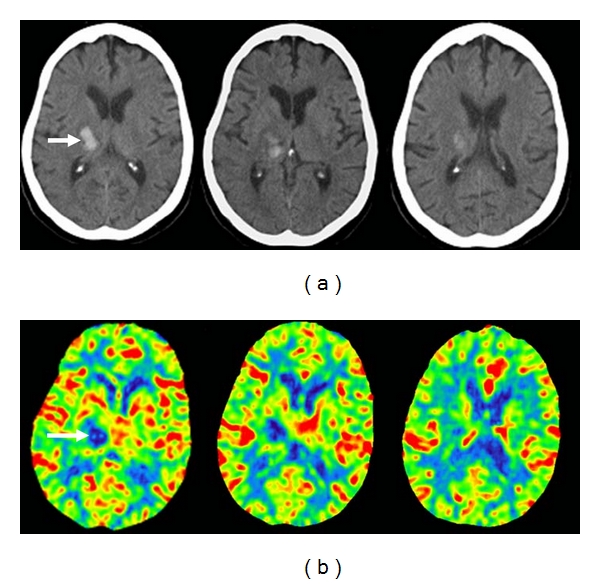
75-year-old woman presented with left hemiparesis and headache. (a) Axial unenhanced CT images show a deep right thalamic hemorrhage (arrow) sparing the caudate nucleus. (b) Cerebral blood flow images show an area of decreased flow matching the area of the hematoma. There are no underlying features to suggest that this is a hemorrhagic infarct.

**Figure 2:**
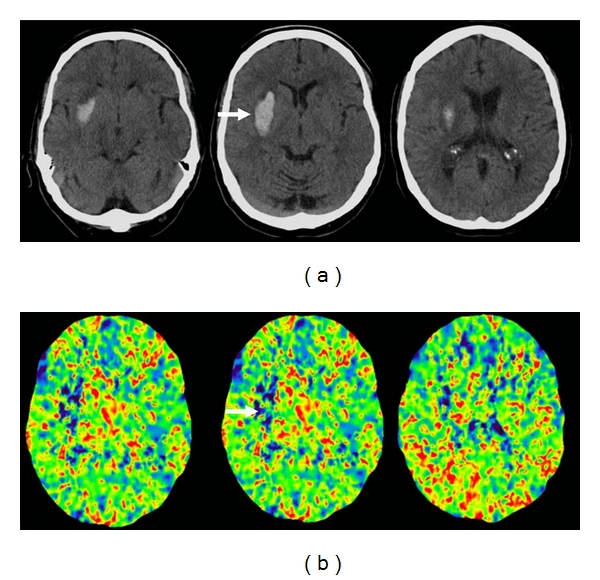
67-year-old man presented with left-sided hemiparesis. (a) Axial unenhanced CT images 2 hours after stroke show a hematoma lateral to the right lentiform nucleus with minimal surrounding hypodensity (arrow). The caudate nucleus is spared. (b) Similar to Figure 1, there is an area of decreased cerebral blood flow in the area of the hematoma. The overall picture is consistent with intracerebral hemorrhage.

**Figure 3:**
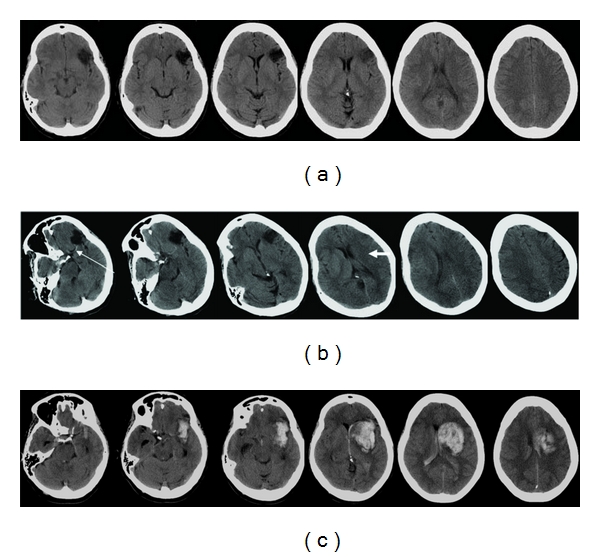
74-year-old woman presented with right hemiparesis lasting few minutes but ongoing residual sensory deficits. (a) Initial axial unenhanced CT images show an old left frontal infarct only. (b) 12 hours after her initial symptoms, she developed recurrent right hemiparesis and aphasia. Repeated axial-unenhanced CT images show obscuration and loss of grey white differentiation in the left lentiform nucleus (thick arrow) and a dense left middle cerebral artery (thin arrow). (c) Axial unenhanced CT images 24 hours after stroke show a large left parenchymal hematoma within the striatocapsular region. Importantly, even in the absence of the previous CT studies, the involvement of the caudate head in the last series of CT images raises the possibility of hemorrhagic infarct. Stage IIH d2 [1].

**Figure 4:**
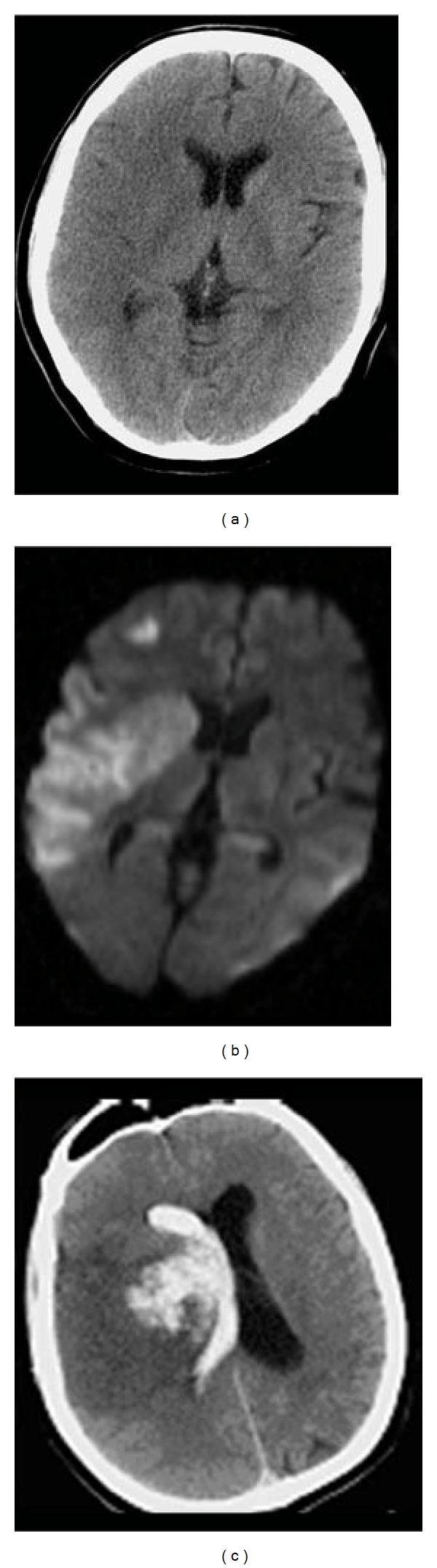
55-year-old man presented with dense left hemiparesis one week after right carotid stenting. (a) Initial axial unenhanced CT shows a large area of low attenuation in the right middle cerebral artery territory. (b) axial DW-MR image 2 hours later confirms a large infarct in the striatocapsular region, including the right caudate head. (c) Axial unenhanced CT 12 hours later shows a large parenchyma hemorrhage within the wedge-shaped area of infarction, consistent with hemorrhagic transformation. The case demonstrates two features typical of hemorrhagic infarct: the involvement of the caudate head and the distribution of the oedema surrounding the stroke lesion following the affected arterial territory. Stage IIH d2 [1].

**Figure 5:**
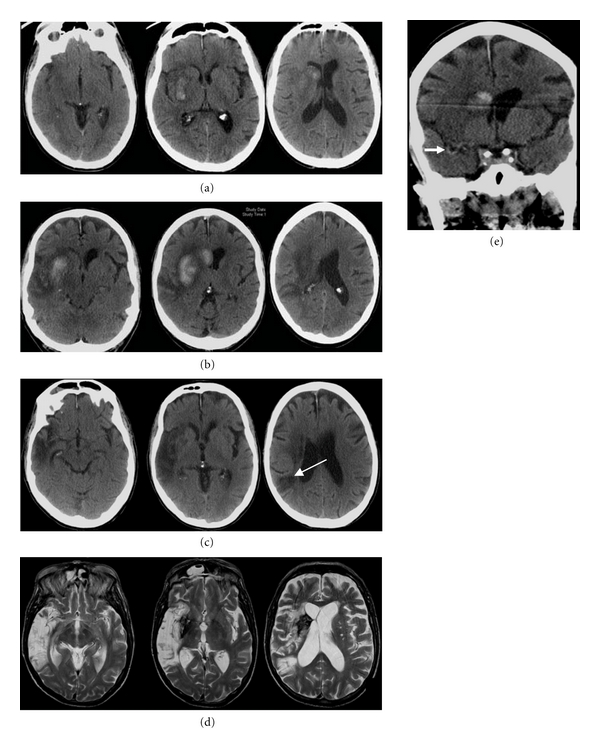
69-year-old man with atrial fibrillation on warfarin, he presented with a 2-day history of left hemiparesis and neglect. (a) Initial axial unenhanced CT images show hemorrhage in the right basal ganglia, involving the right caudate head, unusual for ICH. Stage IIH d1 [1]. Warfarin was stopped and anticoagulation was reversed. (b) Axial unenhanced CT images 10 days later show further extension of hematoma with the surrounding hypodensity extending out to the cortex. (c) Axial unenhanced CT images 1 month after stroke show an extensive area of hypoattenuation in the right MCA territory. There is also an area of low attenuation more posteriorly (thin arrow), not evident on the previous CT and separate from the initial stroke, raising the possibility of a new subacute infarct since the first stroke. (d) Axial T2-weighted MR images 3 months after stroke show the posterior temporal infarct evident on the last CT scan more clearly, suggestive of bland infarction in that region. The cessation of warfarin after the first stroke probably contributed to this cardioembolic stroke. (e) On reviewing the initial CT images, a dense right middle cerebral artery sign is present on the coronal view (but not axial), further suggesting the first stroke is a hemorrhagic infarct rather than a hemorrhagic stroke.

**Figure 6:**
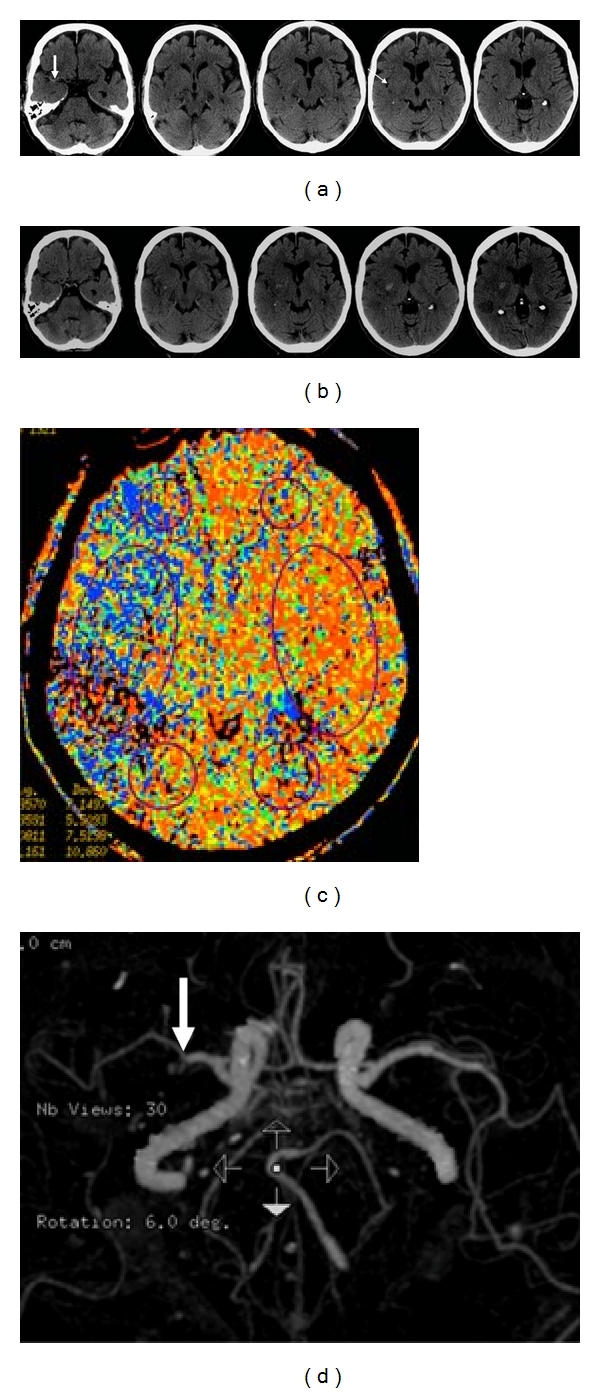
82-year-old man presented with left hemiparesis. (a) Axial unenhanced CT images 2 hours after stroke show a hyperdense right MCA (thick arrow) and loss of the insular ribbon (thin arrow). Patient received intravenous thrombolysis after the CT. (b) Axial unenhanced CT images 3 days later show petechial hemorrhages in the striatocapsular area and a hematoma within the right lentiform nucleus, consistent with hemorrhagic transformation after thrombolysis. Stage HI d1 [1]. (c) CT perfusion image shows a large area of delayed mean transit time on the right. (d) CT angiogram shows truncation of mid M1 segment of the right middle cerebral artery (arrow).

ICH involving the caudate nucleus is uncommon [12, 13] (Figures 1 and 2) and involvement of both the caudate nucleus and putamen may suggest embolism affecting the lenticulostriate arteries and hemorrhagic infarction of the striatocapsular region (Figures 3, 4, and 5). Petechial hemorrhage after intravenous thrombolysis is easily recognized given there is always a baseline CT scan done prior to thrombolysis. The initial CT scan may also show coexisting signs of ischaemia such as the hyperdense middle cerebral artery (MCA) sign and the loss of insula ribbon (Figure 6). 

**Figure 7:**
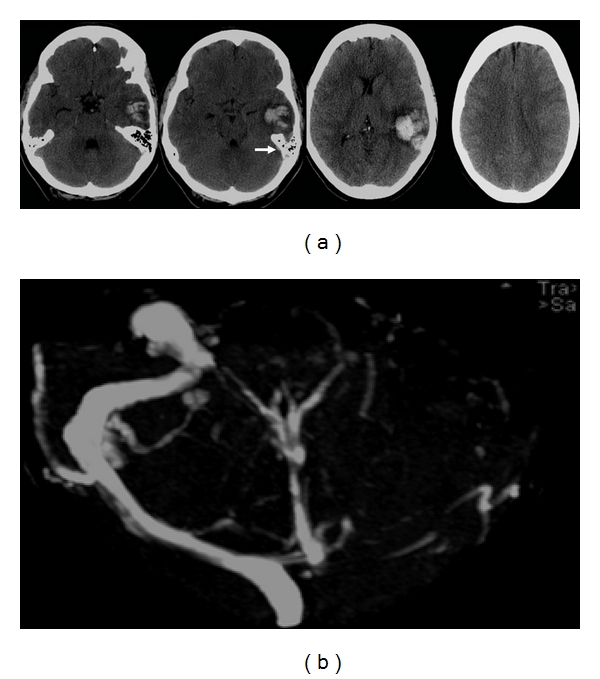
43-year-old woman presented with acute confusion with no history of trauma. (a) Axial unenhanced CT images show a large hemorrhage centered in the left temporal lobe. The subtle hyperdensity in the left sigmoid sinus is suggestive of thrombus (arrow). The topography of the lesion is not what would be expected from a hemorrhagic infarct involving the inferior division of the middle cerebral artery, that is, the surrounding hypodensity fails to reach the cortical surface. (b) MR venogram shows occlusion of the left transverse and sigmoid sinuses, confirming the hemorrhage is secondary to venous infarction.

**Figure 8:**
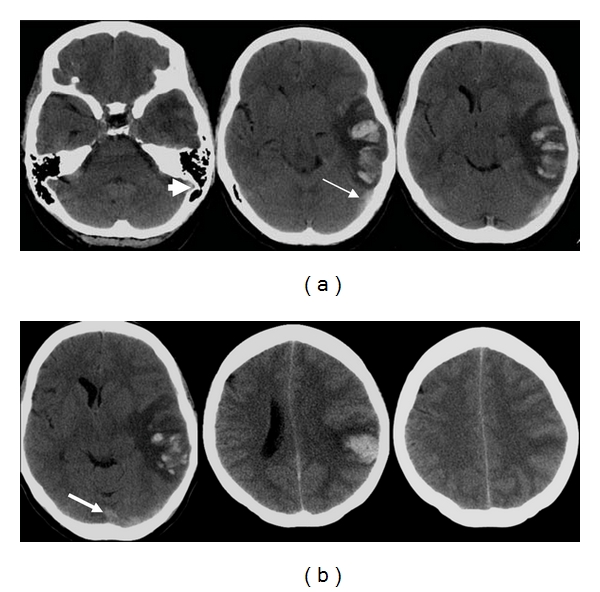
57-year-old woman presented with dysphasia, headache, and vomiting. Axial unenhanced CT images show hemorrhage in the left temporal parietal area. Apart from the typical temporal location suggesting this may be a venous hemorrhage, there is also high attenuation in the left sigmoid sinus (arrowhead), left transverse sinus (thin arrow), and straight sinus (thick arrow). MR venogram confirms occlusion of these sinuses (not shown).

**Figure 9:**
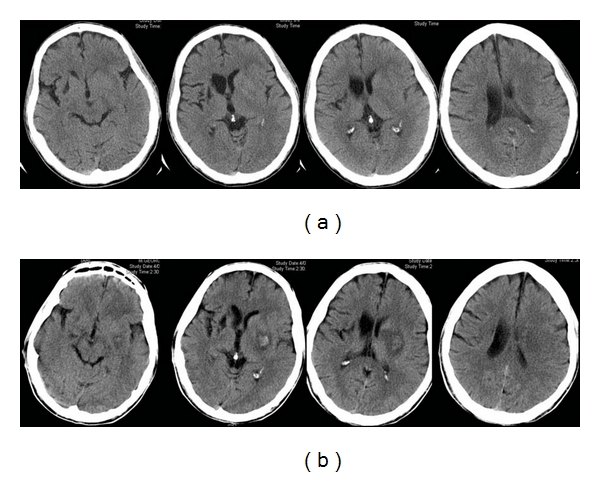
78-year-old man presented with right hemiparesis and dysphasia. (a) Axial unenhanced CT images 5 hours after stroke show an area of low attenuation in the left lentiform nucleus. (b) Repeated axial unenhanced CT images 3 days later show a parenchymal hematoma within the area of infarct. Stage HI d2 [1]. Even if the initial CT images are not available, the topography of the stroke is suspicious for hemorrhagic infarct. The centre of the hematoma is in the striatocapsular region with the surrounding hypodensity extending superiorly, following the topography of the middle cerebral artery.

**Figure 10:**
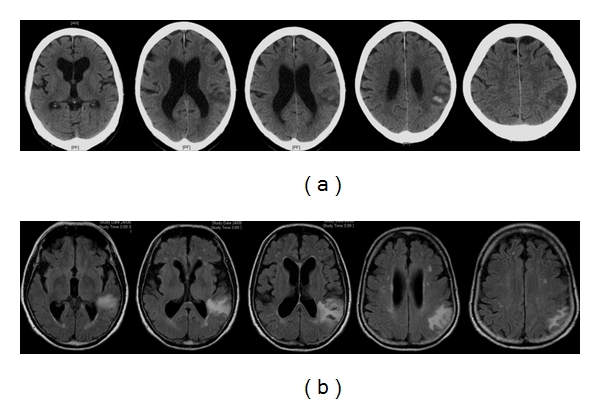
75-year-old man presented with slurred speech. (a) Axial unenhanced CT images show an acute left parietal hemorrhage. The hypodense area around the hemorrhage reaches superiorly and out to the cortical surface, following the middle cerebral artery territory. Stage HI c2 [1]. (b) Axial fluid-attenuated inversion recovery MR sequences confirm the area of infarction reaching the surface of the cortex, suggesting that the stroke is a hemorrhagic infarct.

**Figure 11:**
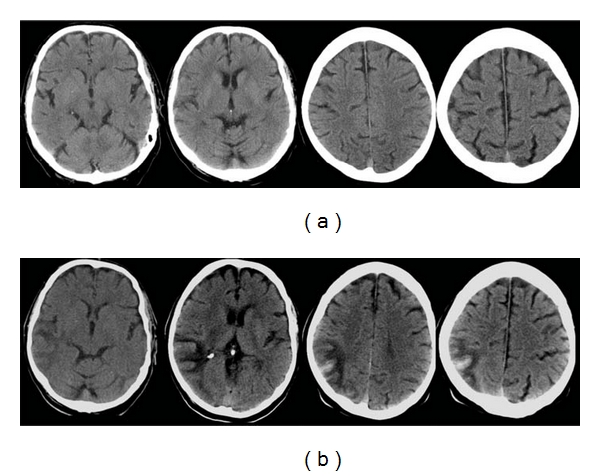
83-year-old man with atrial fibrillation, he presented with left-sided weakness and neglect. (a) Axial unenhanced CT images within 3 hours of symptom onset show no acute changes. (b) Axial unenhanced CT images 10 days after stroke show a hemorrhage within a wedge-shaped infarct in the right posterior parietal lobe. The surrounding hypodense area follows the topography of the middle cerebral artery, reaching out to the cortex and superiorly, consistent with a hemorrhagic infarct. Stage HI c2 [1].

The hypodense region of oedema surrounding the hematoma in ICH usually radiate centripetally, and it does not follow the topography of an arterial territory. Similar pattern of oedema is seen in hemorrhages resulting from venous infarction (Figures 7 and 8). In patients with HI, the hypodense regions surrounding the hematoma may reach the cortical surface and spread far from the centre of the hematoma (Figures 9, 10, and 11). The topography of this hypodense region usually follows the affected vascular territory. Maps of the MCA [14] and the posterior cerebral artery (PCA) infarct territory [15] have been recently published and can be used to aid assignment of territorial membership of the stroke. The centre of the hematoma in cases of HI seems to correspond to regions at highest risk of infarction on the infarct map. For example, in the MCA territory, the region at risk is the striatocapsular region and in the PCA territory, the medial temporal and occipital lobes.

## 4. MR Imaging Features of HI

**Figure 12:**
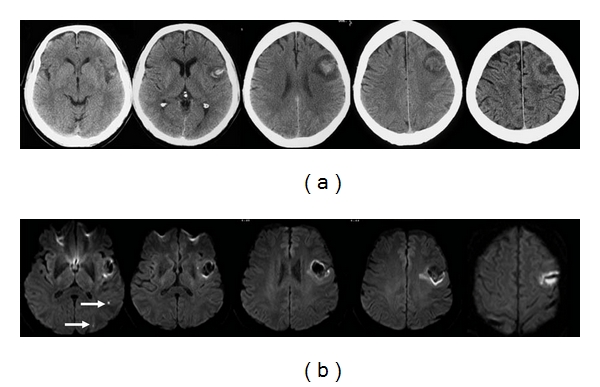
60-year-old man presented with dysphasia and confusion for 2 days. (a) Axial unenhanced CT images show a left frontal hematoma with surrounding hypodensity spreading from the centre, reaching superiorly and out to the cortical surface. The shape and topography of the lesion suggest that the primary event is an infarct, with secondary hemorrhagic transformation. Stage HI c2 [1]. (b) Axial diffusion weight MR images show 2 small discrete lesions within the left parietal and temporal lobes, suggesting concurrent infarcts in the same arterial territory. This further supports that the initial lesion is a hemorrhagic infarct, probably embolic in nature.

**Figure 13:**
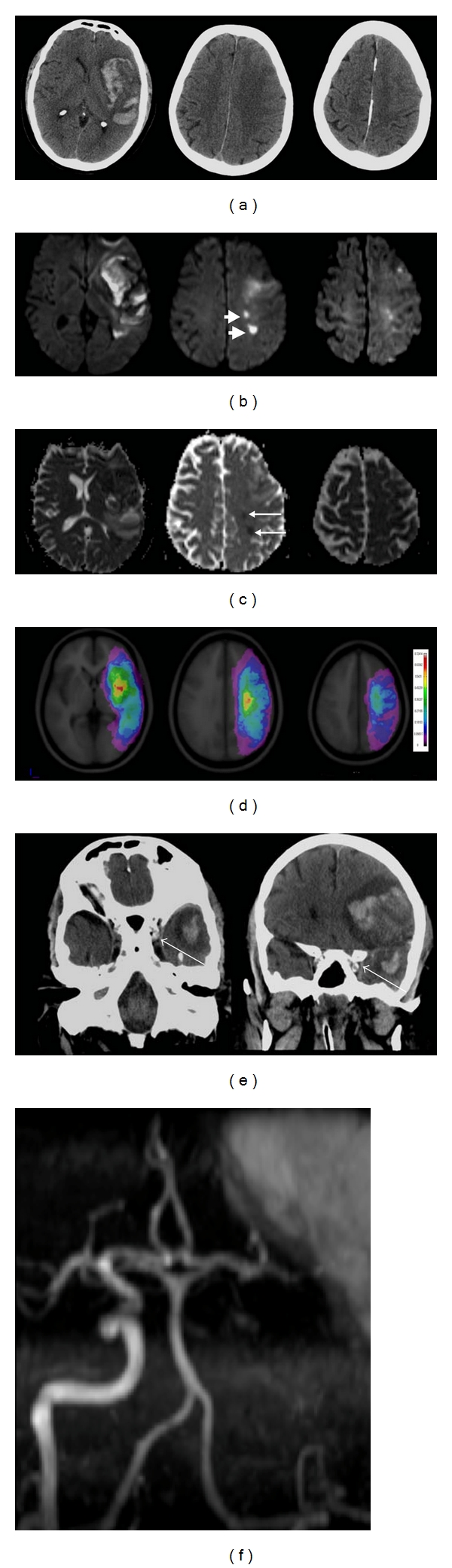
54-year-old man with a history of idiopathic thrombocytopenic purpura presented with acute coronary syndrome. He developed a dense right hemiparesis overnight. (a) Initial axial unenhanced CT images show a large left “fronto-temporal hemorrhage”, initially thought to be secondary to his low platelet count of 20. Stage IIH c2 [1]. (b) Axial diffusion weighted MR images reveal areas of restricted diffusion remote from the area of hemorrhage (arrow heads). (c) The areas of diffusion weighted abnormality have low apparent diffusion coefficient values (arrows). (d) Digital probabilistic maps of middle cerebral artery territory infarcts show both the infarcts and hemorrhage lie within the middle cerebral artery territory. (e) Coronal unenhanced images from the original CT show a dense left internal carotid artery in the cavernous sinus (thin arrow). (f) MR angiogram confirms occlusion of the left internal carotid middle cerebral arteries. The overall picture suggests that the stroke is a HI.

**Figure 14:**
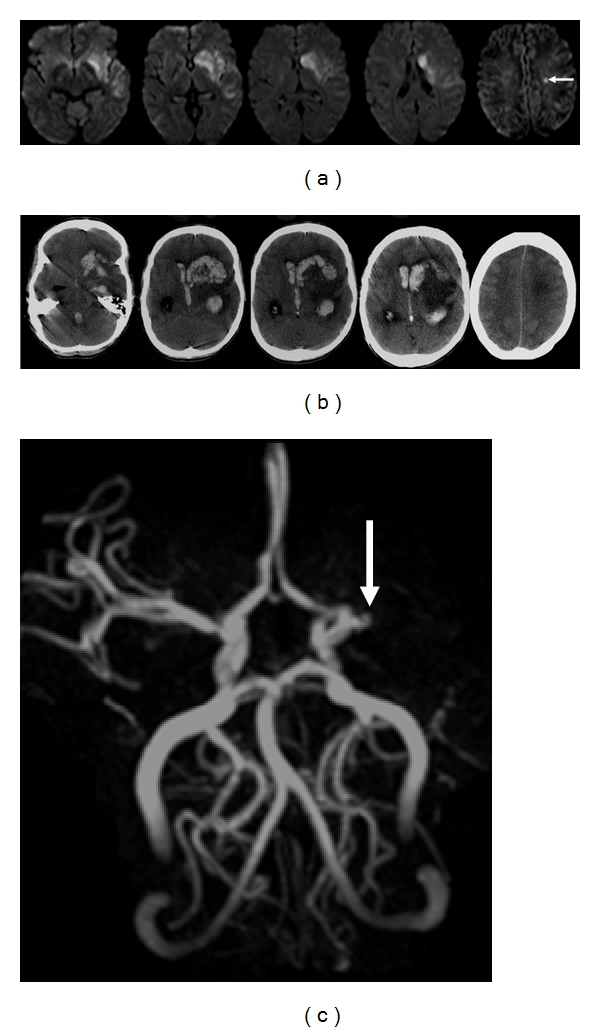
24-year-old woman presented with dense right hemiparesis and seizure. (a) Initial axial diffusion weighted MR images show an extensive area of diffusion restriction in the left basal ganglia and insular cortex. There is also a small area of restricted diffusion in the left corona radiate (arrow). Staphylococcus was grown from her peripheral blood culture, and she was treated for bacterial endocarditis. (b) Axial unenhanced CT images 3 weeks later show extensive hemorrhage within the area of the initial infarction. Stage IIH d2 [1]. (c) MR angiogram shows occlusion of the left middle cerebral artery, consistent with hemorrhagic transformation of the initial stroke.

The magnetic resonance (MR) imaging features of HI on diffusion weighted imaging (DWI) sequence have a mixed appearance. Within the hemorrhagic area, the appearance between HI and ICH is indistinguishable. However, the presence of an ischaemic process may be evidenced by discrete regions of restricted diffusion remote from the hemorrhagic area (Figures 12–14). These lesions further strengthen the possibility of the primary lesion being a HI.

Time-of-flight MR angiography can show the presence of occlusive intracranial disease and hence aids in confirming the diagnosis of HI (Figures 13-14). Although not widely available, MR perfusion imaging may help in diagnosing HI if it shows the presence of a perfusion deficit extending beyond the region of hematoma. In ICH, the region of perfusion deficit does not extend beyond the ICH [16].

The presence of “microbleeds” on gradient-echo (GRE) or susceptibility weighted imaging (SWI) sequence suggests the presence of blood product but does not necessarily indicate that the lesion in question is HI or ICH [17]. In elderly patients, it has been recognised that some patients with ischaemic stroke may also have evidence of silent microbleeds. Lobar ICH tend to be located posteriorly, corresponding to the distribution of microbleeds and the location of binding of amyloid tracer in PET studies [18].

## 5. Role of CT Angiography and Perfusion

CT angiography (CTA) is often used as a screening tool to exclude the possibility of aneurysmal bleed. It can also be used to concurrently evaluate the possibility of arterial occlusion and potential intra-arterial therapy. Given the additional risk of radiation exposure and iodinated contrast agents, further studies are required to evaluate the usefulness of this modality for determining arterial occlusion in patients with isolated putaminal or thalamic hemorrhage.

CT perfusion (CTP) with cerebral blood flow, cerebral blood volume, and mean transit time is usually performed at the same time as CTA in tertiary stroke centres. When this is available, it can help with differentiation between HI and ICH. In contrast to ischaemic stroke, a large perfusion defect around an ICH has not yet been reported. The presence of such a mismatch may point to the possibility of HI.

## 6. Conclusion

Differentiating HI from ICH can be difficult. Careful examination of the topography of the stroke on the initial CT in different sections may distinguish the two conditions. Signs compatible with an infarct such as dense artery sign and insular ribbon sign should be actively looked for. Advanced imaging technique such as CTA, CTP, and MR imaging may be particularly helpful in difficult cases, looking for perfusion deficit, arterial occlusion, and diffusion restriction remote from the site of hemorrhages. Distinguishing HI from ICH is important given the difference in acute and long term management.
